# Importance of Viral Late Domains in Budding and Release of Enveloped RNA Viruses

**DOI:** 10.3390/v13081559

**Published:** 2021-08-06

**Authors:** Lisa Welker, Jean-Christophe Paillart, Serena Bernacchi

**Affiliations:** Architecture et Réactivité de l’ARN, UPR 9002, IBMC, CNRS, Université de Strasbourg, F-67000 Strasbourg, France; l.welker@ibmc-cnrs.unistra.fr (L.W.); jc.paillart@ibmc-cnrs.unistra.fr (J.-C.P.)

**Keywords:** viral assembly, budding, late assembly domains, retrovirus, RNA enveloped viruses, retroviral gag precursor, structural viral proteins, ubiquitination, ESCRT machinery

## Abstract

Late assembly (L) domains are conserved sequences that are necessary for the late steps of viral replication, acting like cellular adaptors to engage the ESCRT membrane fission machinery that promote virion release. These short sequences, whose mutation or deletion produce the accumulation of immature virions at the plasma membrane, were firstly identified within retroviral Gag precursors, and in a further step, also in structural proteins of many other enveloped RNA viruses including arenaviruses, filoviruses, rhabdoviruses, reoviruses, and paramyxoviruses. Three classes of L domains have been identified thus far (PT/SAP, YPXnL/LXXLF, and PPxY), even if it has recently been suggested that other motifs could act as L domains. Here, we summarize the current state of knowledge of the different types of L domains and their cellular partners in the budding events of RNA viruses, with a particular focus on retroviruses.

## 1. Introduction

Viruses are ubiquitous obligate intracellular parasites multiplying only within living cells by usurping the cellular machinery of their host to produce progeny virions. They are composed of extremely diverse nucleoprotein entities, classified into DNA or RNA viruses according to the type of nucleic acid constituting their genome. Even though DNA and RNA viruses are likely to infect both prokaryotes and eukaryotes, the two different classes are not found with the same abundance. Indeed, while the prokaryotic virosphere is dominated by DNA viruses (especially with double-stranded DNA genome), eukaryotes are rather found hosting a wide variety of RNA viruses (for a review, see [1,2]). A considerable number of those RNA viruses are responsible for severe human diseases, and thus represent a serious threat to global health. Since pathogenic RNA viruses are widely involved in zoonoses and can quickly adapt to the new host, they are considered as major etiological agents of emerging infectious diseases in humans (for a review, see [3]). Indeed the pandemic caused by influenza A (H1N1) in 1918, which led to more than 50 million deaths worldwide, was followed during this last century by many other epidemics and pandemics caused by RNA viruses, that include human immunodeficiency virus (HIV-1), identified in 1983 as the causative agent of the acquired immunodeficiency syndrome (AIDS), which has caused an estimated 34.7 million deaths according to UNIAIDS [4]; ebolaviruses, causing systemic disease with high lethality rate; and also coronaviruses, associated with severe respiratory illness, such as the severe acute respiratory syndrome (SARS) and Middle East respiratory syndrome coronavirus (MERS). Most recently the SARS coronavirus 2 (SARS-CoV-2) emerged in 2019, having infected more than 178 million people by mid-June 2021 and continuing to spread around the world. In this context, the surveillance and the biological study of RNA viruses are essential tools to control their spread and the diseases they cause. 

**Figure 1:**
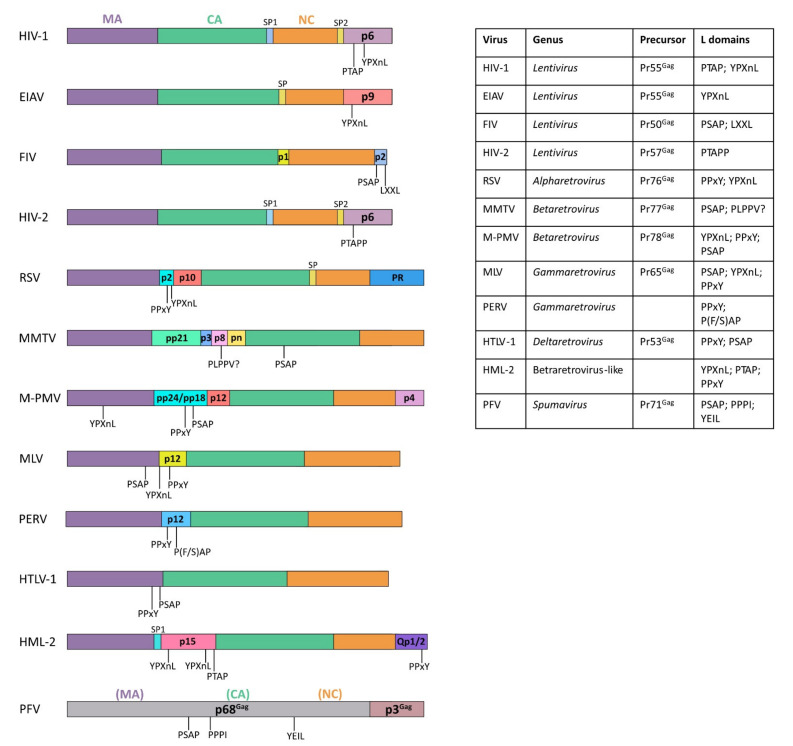
Retroviral Gag organization domains. All retroviruses display matrix domain (MA, in violet), capsid (CA, in green), and nucleocapsid (NC, in orange). Spacer peptides (SP) and peptide sequences containing L domains are indicated within structural precursors of HIV-1, EIAV, FIV, HIV-2, RSV, MMTV, M-PMV, MLV, PERV, HTLV-I, HML-2, and PFV.

Viral dissemination relies on an extracellular phase in the virus life cycle. Indeed, the progeny virions formed in infected cells must be released and enter new target cells, therefore overcoming the barriers formed by cellular membranes and cytoskeleton. To this aim, viruses use three main strategies for egress: cell lysis, budding, and exocytosis. Non-enveloped RNA viruses (also termed as “naked viruses”) are generally thought to exit infected cells by lysis, thereby causing disruption of cellular membranes. However, there are some exceptions to this rule, and it has been demonstrated that viruses such as hepatitis A virus, poliovirus, rotavirus, and norovirus can exit cells non-lytically within vesicles, thus enclosing themselves into host-derived membranes [5,6,7]. As this feature is usually considered as the major difference between enveloped and non-enveloped viruses, viruses using this strategy for cellular egress are sometimes defined as “quasi-enveloped” (see Section 7). The budding process is commonly employed by enveloped RNA viruses that derive their envelopes enriched by viral glycoproteins, directly from the host. Budding of many RNA viruses, including retroviruses, orthomyxoviruses, filoviruses, alphaviruses, and rhabdoviruses, occur at the plasma membrane (PM), and in this case, progeny virions are directly released in the extracellular environment. For other enveloped RNA viruses, including coronaviruses, flaviviruses, and bunyaviruses, budding occurs at intracellular membranes into the lumen of organelles such as the endoplasmic reticulum (ER), the Golgi apparatus, the ER–Golgi intermediate compartment (ERGIC), and the endosomes. In this case, virus egress relies on the cellular secretory pathway, and the viral budding process is mechanistically equivalent at the PM and at cellular organelles, since in both cases it implies the deformation of the membrane during the viral particle envelopment process. This event is then followed by membrane fission, thus allowing the release of viral particles and the generation of two distinct cellular and viral membranes. Importantly, membrane fission is achieved by hijacking the endosomal sorting complexes required for transport (ESCRT) pathway (for a review, see [8] and Section 6). These cellular factors comprise three subcomplexes (ESCRT-I, ESCRT-II, ESCRT-III), as well as the ESCRT-III-associated ALG-2-interacting protein X (ALIX) and the ATPase vacuolar protein sorting-associated 4 (VSP4). The early acting ESCRT complexes (ESCRT-I and ESCRT-II) assemble stably within the cytoplasm and can associate with adaptor proteins such as the HRS/STAM complex (also named ESCRT-0) to recruit and activate late-acting ESCRT-III and VSP4 factors at specific membrane sites where the assembling virions and fission events occur (for reviews, see [9,10,11]). Viral assembly and the recruitment of the ESCRT machinery must thus be coordinated in space and time, and it is therefore not surprising that these steps are finely orchestrated by viral structural proteins. Indeed, these multifunctional proteins were found to recruit the ESCRT machinery at viral budding sites via conserved motifs, termed late assembly domains (L domains) that act like cellular adaptor proteins (for a review, see [12]). Historically L domains were first identified within Gag (group-specific antigen) structural precursors of retroviruses (Figure 1) as HIV-1, Rous sarcoma virus (RSV) and equine infectious anemia virus (EIAV), HIV-2, feline immunodeficiency virus (FIV), human T cell leukemia virus type 1 (HTLV-1), murine leukemia virus (MLV), porcine endogenous retrovirus (PERV), human endogenous retrovirus-K (HML-2), Mason–Pfizer monkey virus (M-PMV), and prototypic foamy viruses (PFV) [13,14,15,16,17,18,19,20,21,22,23,24,25]. The different retroviral Gag proteins are ubiquitinated, and importantly, ubiquitin was observed to be linked to the budding machinery (see Section 6) [26,27,28,29].

**Figure 2:**
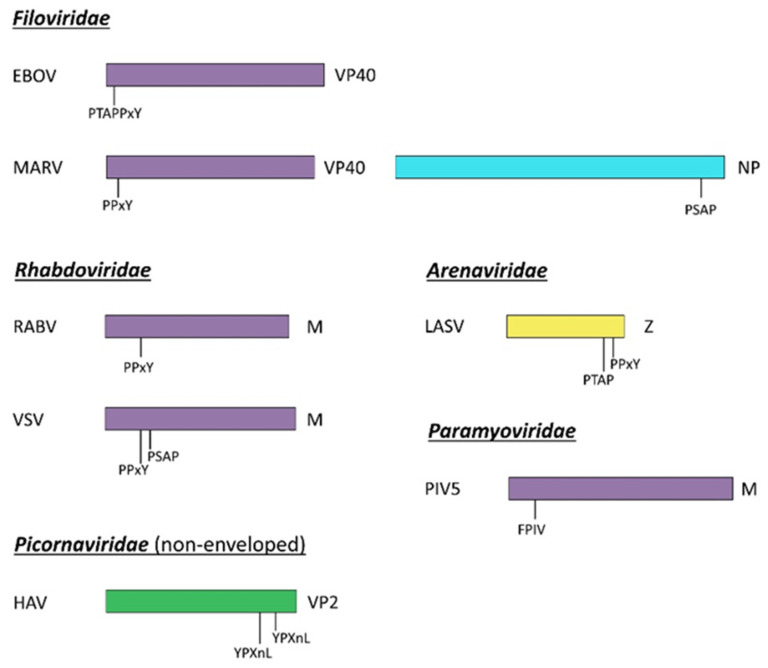
L domains in the structural proteins of RNA viruses including filoviruses (EBOV and MARV), rhabdoviruses (RABV and VSV), arenaviruses (LASV), paramyoviruses (PIV5), and picornavirus (HAV).

In a further step, L domains were also found in structural proteins of many other RNA enveloped viruses, including arenaviruses, filoviruses, rhabdoviruses, reoviruses, and paramyxoviruses (Figure 2).

Of note, some enveloped RNA viruses such as influenza A [30,31] were found to not rely on L domains, and thus cellular egress is regulated by ESCRT-independent budding mechanisms, as extensively described in [32].

This review relates the history of L domains discovery and aims to provide a current state of knowledge concerning the function of L domains and their cellular partners involved in the budding events of RNA viruses, with a particular focus on retroviruses. Considering the importance of budding process in the viral life cycle, a better comprehension of molecular mechanisms driving this step would constitute an asset to the development of new antiviral drugs that could be effective against multiple viruses.

## 2. Identification of L Domains within HIV-1 p6 Domain of Pr55^Gag^ Precursor

**Figure 3:**
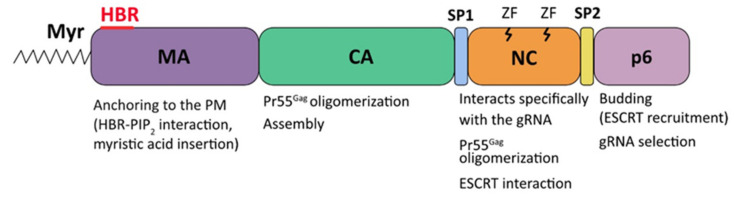
Schematic representation of the domains in HIV-1 Pr55^Gag^ precursor. The myristoyl moiety is indicated at the N-terminus of the precursor. The MA domain drives the interaction between the Pr55^Gag^ and the PM through a bipartite signal consisting of a HBR domain and the covalently attached myristic acid moiety. The CA domain of Pr55^Gag^ mediates Pr55^Gag^ oligomerization and ensures formation of the core of the mature viral particles, and the NC domain, which contains two zinc-finger motifs, corresponds to the primary binding motif to nucleic acids, and contributes to the Pr55^Gag^ multimerization. The p6 domain contributes to the specificity of Pr55^Gag^–gRNA interactions and is essential for viral budding. Pr55^Gag^ contains also two spacer peptides which are SP1 and SP2.

All retroviruses express structural Gag polyproteins that drive the assembly and the release of virus-like particles (VLPs), even in the absence of other viral components (for reviews, see [33,34]), and all retroviral Gag precursors contain the three structural domains of matrix (MA), capsid (CA), and nucleocapsid (NC), each of them playing an essential role in the retroviral particle assembly and in budding processes. Gag usually also carry additional domains and spacer peptides such as, for example, the HIV-1 p6 domain, as well as SP1 and SP2 linkers (for reviews, see [34,35,36,37]) (Figure 3).

Amongst retroviral precursors, the role of the different domains of HIV-1 Gag (also named Pr55^Gag^) in the encapsidation of gRNA into the viral particles was extensively characterized [34,35,36,37,38,39] (Figure 3). The HIV-1 assembly process occurs at the PM, where about 2000 copies of Pr55^Gag^ accumulate to produce immature particles. The targeting of Pr55^Gag^ polyproteins to lipid rafts at the PM [40] is driven by the MA domain at the N-terminus of the precursor via a bipartite signal. The latter is composed by a myristic acid moiety, and a highly basic region (HBR) located on the surface of MA domain, which forms electrostatic interactions with negatively charged phosphatidylinositol-(4,5)-bisphosphate (PIP_2_) that are specifically enriched at the inner leaflet of PM [41]. The myristic acid is initially sequestered within the MA domain to prevent unspecific interactions with cellular internal membranes. In a further step, the interactions between the HBR and PIP_2_ at PM, in parallel with Pr55^Gag^-Pr55^Gag^ interactions, lead to the exposure of the myristic acid moiety [42,43,44], which is then inserted into the PM, thus allowing the anchoring of the precursor by hydrophobic interactions. The CA domain mediates the oligomerization of Pr55^Gag^ and drives the immature particle assembly process. Then, the SP1 spacer, which is located between CA and NC domains, contributes to Pr55^Gag^ multimerization and acts as a molecular switch to initiate the assembly of immature VLPs [45,46,47,48]. The NC domain contains two zinc-finger motifs (ZFs) and interacts specifically with the dimeric viral RNA genome (gRNA) to ensure its encapsidation within the virions [49,50,51,52,53,54,55], and the SP2 domain that separates the NC and the C-terminus p6 domain plays a role in Pr55^Gag^ processing, even though the deletion of this domain has only a minor effect on viral infectivity, and did not induce major alterations in mature core morphology [56]. Finally, the p6 domain seems to mediate specific interactions occurring between Pr55^Gag^ and gRNA [57] and regulates the budding of viral particles at the PM by recruiting the cellular factors of the ESCRT pathway. Indeed, mutations in this domain completely inhibited the budding of virions from the PM of COS-7 cells [13]. In particular, truncated forms of Pr55^Gag^ lacking the p6 domain were found to be able to assemble into VLP, even though, as shown by electron microscopy, they are not released into extracellular medium and remain attached to the cell membrane by a thin tether [13]. All together, these observations provided the first evidence that retroviruses code for regions specifically required for virus release. Thereafter, extensive mutagenesis analyses of the p6 domain showed that a PTAP motif is crucial for the release of viral particles [58,59]. Subsequently, P(T/S)AP motifs (where the second residue can be either a Ser or a Thr) were also identified within structural proteins of others retroviruses, filoviruses, rhabdoviruses, and arenaviruses (for a list, see [8]), and at least two other conserved motifs (PPxY and YPXnL) displaying similar functions in efficient viral budding were identified within retroviral Gag proteins, as a number of studies have demonstrated their interactions with components of the ESCRT machinery (for a review, see [60]). All these short sequences were thus termed “L domains” to emphasize their role in the late phases of the assembly process during the separation of virions from the cell membrane.

In the specific HIV-1 context, both PTAP and YPXnL motifs within the p6 domain were observed to promote the viral egress by interacting with the ESCRT machinery, as the PTAP domain was found to be bound by the ESCRT-I tumor suppressor gene 101 (TSG101) [61] and the YPXnL domain by the ESCRT-III associated ALIX protein [62] (see Section 6.1 and Section 6.2).

In Pr55^Gag^, in addition to p6, the NC domain was also found to play a role in viral release as the latter participates in the recruitment of the ESCRT cellular proteins necessary for PTAP- and YPX(n)L-mediated budding [63]. Indeed, the deletion of the NC domain decreased in cells the amount of the complexes formed by Pr55^Gag^ and TSG101 [64]. Similarly, mutation of NC resulted in the release of DNA-containing viruses, which was the consequence of budding defects mainly associated with deficiency in the recruitment of TSG101 [65]. Moreover, the interactions between NC and the ESCRT-III-associated factor ALIX were found to be promoted by ZFs, as mutations of these motifs resulted in defective production of viral particles [66]. Interestingly, TSG101 was found to substitute for the distal ZnF2, since chimeric Gag-ΔZF2-TSG101 were found to rescue budding [67], thus confirming the role of those motifs in the recruitment of ESCRT machinery.

## 3. Identification of the PPxY L Domain within RSV p2b Domain of Pr76^Gag^

Mutagenesis analysis on the Gag polyprotein (Pr76^Gag^) of the avian alpha-retrovirus RSV revealed that the p2b spacer peptide located between the MA and CA domains is necessary for the release of viral particles [14] (Figure 1). In line with this idea, truncation of the p2b peptide caused severe release defects, showing a phenotype analogous to what was observed for HIV-1 p6 mutants in which viral budding was arrested at the last stage [13]. The function of p2b in virus release was then attributed to a Pro-rich L domain that is located between the MA and the CA domains. This L domain was later finely mapped to the highly conserved amino acid sequence PPPPYV, and defects caused by its deletion can be rescued by its replacement at several other positions in RSV Gag [68]. Moreover, this RSV L domain was observed to specifically interact with the E3 ubiquitin protein ligases NEDD4-like family ([69], and see Section 6.3). In a further step, these PPxY L domains (where x could be any residue, even if it is often a Pro) were also found in several other viruses [8], except for Gag proteins of lentiviruses including HIV-1 [14]. Interestingly, by using RSV Gag chimeras, researchers showed that the L domain activity carried by p2b could be functionally replaced with the C-terminal HIV-1 p6 sequence, and these domains could function independently of their position in the precursor since they can be located either internally or at the C-terminus [15]. Thus, these findings suggested for the first time that, despite their differences, L domains share the same function as they both ensure the release of viral particles.

## 4. Identification of the YPXnL L Domain within the EIAV p9 Domain of Pr55^Gag^

Similarly to the HIV-1 p6 domain, the p9 domain located at the C-terminus of the EIAV Gag protein (named EIAV Pr55^Gag^) (Figure 1) was found to rescue the RSV viral particle release defect induced by p2b deletion, thus suggesting the presence of a L domain in p9 [15]. Interestingly, unlike other lentiviruses, the EIAV p9 domain does not contain a P(T/S)AP L motif, and moreover, no sequence homology between EIAV p9 and its functional homologues RSV p2b and HIV-1 p6 was identified. Thereafter, mutagenesis analysis revealed that substitutions of each residue Y23, P24, or L26 with A within p9 abolished viral release, while other mutations within the same domain did not display any effect on this step. This analysis demonstrated that the p9 L domain activity is supported by a YPXL motif [16], which was then identified in several other viruses [8] in the more generic YPXnL form (where Xn can vary in amino acid sequence and length). Remarkably, budding assay of EIAV/HIV-1 and EIAV/RSV chimeric Gag polyprotein demonstrated that p6 and p2b, respectively, can replace the EIAV p9 domain. These data, combined with similar observations on the substitution of the p2b domain of RSV precursor by p6 and p9 domains [15], confirmed the notion these L domains are mostly interchangeable.

## 5. Functional Exchangeability and Multiplicity of L Domains

The functional exchangeability of L domains has indeed been widely demonstrated in several viral contexts [15,16,70,71,72,73,74]. In addition to HIV-1, EIAV, and RSV, similar experiments have also been carried out with gamma-retrovirus. The MLV Gag polyprotein (Pr65^Gag^) (Figure 1) indeed contains a PPPY L motif in its p12 domain, located between the MA and CA domains, similarly to RSV. Interestingly, in a PPPY-defective MLV Pr65^Gag^ protein, the insertion of the p12 domain containing the PPPY motif and its flanking sequences at different positions within the precursor can partially or completely restore the release of viral particles. Moreover, in these same PPYP-deficient Pr65^Gag^, insertion of the RSV PPYP L domain or the HIV-1 PTAP domain within the MA domain of MLV Pr65^Gag^ was found to rescue the assembly defects [70]. In addition, L domains were found to be functionally exchangeable also with viruses outside the *Retroviridae* family. The first description of L domains and their function in other viral families was reported in Rhabdovirus [71], where a PPxY conserved motif with analogous properties to those observed for the L domain in RSV p2b, was identified in matrix (M) of Rabbies virus and of vesicular stomatitis virus (VSV) [75] (Figure 2). Notably, the N-terminal part of the VSV M protein, which includes the PPxY motif, was found to replace the MA-p2b region of RSV Pr76^Gag^, and since mutations in this motif severely impaired the budding of the M-Gag chimera, this PPxY domain was assigned as the VSV L domain [71]. Similarly, the L domain of Ebola virus (EBOV), which consists in an overlapping of P(T/S)AP and PPxY motifs within the viral VP40 protein (Figure 2), can restore the budding of a minimal assembly system in which HIV-1 Gag lacks the natural NC-p1 and p6 domains [76].

Overall, the regulation of viral release mediated by L domains is highly conserved among different groups of viruses, and new motifs acting as L domains were recently identified using the ability of the L domains to be mostly functionally interchangeable. That was, for example, the case of the FPIV motif identified within the M protein of several paramyxoviruses (such as the parainfluenza Virus 5 (PIV5), the mumps virus (MV), and the Newcastle disease virus (NDV)), which was observed to be necessary for viral budding [72,77,78]. Interestingly, when fused to the C-terminal end of HIV-1 Gag proteins lacking the PTAP L domain, the PIV5 FPIV motif was able to partially rescue the budding defect induced by the lack of PTAP [72]. However, mutagenesis experiments suggested a more general sequence O-P-x-V, where the Pro residue was found to be particularly critical for function as its substitution led to poor budding [72]. More recently, a PLPPV motif within the p8 domain of the mouse mammary tumor virus (MMTV) Gag protein (Pr77^Gag^) has been described as a fourth type of retroviral L domain, which can restore the budding defect of EIAV induced by the deletion of the YPXnL domain within the precursor [73]. However, whether this motif is effectively a new type of retroviral L domain is yet to be confirmed, and its precise role in viral budding remains to be fully characterized. Finally, the LXXL motif, which is highly conserved in p2 domain of FIV Pr50^Gag^ precursor (Figure 1), is known to participate to ALIX-mediated release and to interact with clathrin adaptor proteins for budding [18,79,80].

While some viruses such as EIAV rely on one single L domain to ensure their budding, retroviruses such as FIV display two L domains (PSAP and LXXL) within their retroviral precursor. This is indeed a common feature amongst viruses that frequently use a combination of multiple L domains to promote virions egress. For instance, the MLV Gag polyprotein contains three putative late domains (PSAP, LYPAL, and PPPY) located in the MA domain and in p12 (Figure 1) [20]. Similarly, the HIV-1 p6 domain of Pr55^Gag^ contains also a YPXnL motif, which is located downstream to the PTAP domain [62,81]. However, if the PTAP motif is conserved in all subtypes, the HIV-1 subtype C (HIV-1C) naturally lacks the YPX_n_L L domain [82]. Interestingly, in HIV-1C-infected patients in therapy failure, a tetra-peptide PYXE (where X is either Arg, Lys, or Gln) was found to replace YPX_n_L [83]. In this context, this sequence was found to fulfill the role of YPXnL in viral budding in order to increase the replication fitness, as well as to decrease the sensitivity to certain antiretroviral drugs such as the protease inhibitor lopinavir [84,85]. Moreover, viruses such as M-PMV, HTLV-I, EBOV, marburg virus (MARV), and Lassa fever virus (LASV) contain both PPxY and P(T/S)AP L domains (Figure 2) [86,87,88,89,90,91], while others such as RSV contain both YPXnL and PPxY L domains [92]. In the VP40 (or M) protein of EBOV, both domains are required for efficient budding. Interestingly, when these same domains were transplanted in MLV, the two motifs overall contributed to viral budding, while in the HIV-1 context, the PPxY-type L domains were mostly inactive, and L-domain activity was entirely due to PTAP. These context-dependent effects of L-domain function may reflect requirements for distinct host-driven functions during the viral assembly [76,88,93]. Moreover, multiple late domains typically exhibit a hierarchy of importance, considering that mutations in one of the L domains display a more deleterious effect on budding than the other. For instance, in HTLV-1, the viral precursor Pr53^Gag^ carries both PPPY and PTAP L domains (Figure 1), and if PPPY motif cannot be substituted with PTAP or YPXnL domains without affecting viral budding, the replacement of the PTAP domain with either the PPPY or YPXnL motifs has no influence on the release of viral particles [88]. Nevertheless, mutations of either PPPY or PTAP L domains with APPY or PTRP motifs, respectively, impaired viral budding, demonstrating that these L domains do not display redundancy and both play an active role in virus release [88]. Indeed, electron microscopy analysis revealed that mutations in the PPPY L domain of HTLV-1 or of M-PMV cause early budding defects with consequent viral particle accumulation underneath the PM without membrane curvature induction [86,94], while the mutation of P(T/S)AP blocks the release of viruses at a later step [86,95]. Similarly, three different motifs, namely, P(T/S)AP, PPxY, and YPX(n)L, were proposed to play a role in the release of human exogenous retrovirus-K (HML-2). However, further analysis clarified that viral release was predominantly found to be mediated by PTAP motif, as well as by two auxiliary YPX(n)L motifs in the p15 domain of the Gag precursor (Figure 1) [22]. The notion of a hierarchy in L domains function was also supported by the case of the porcine endogenous retrovirus (PERV). Indeed even though its Gag protein contains the two L domains PPPY and P(F/S)AP (Figure 1), the dominant L domain involved in PERV release was found to be PPPY [21]. Taken together, these finding indicate that L domains exhibit distinct functions for viral egress. Of note is the case of the PFV, for which three potential L-domain motifs (PSAP, PPPI, and YEIL) were proposed, considering the homology of these sequences with other viral L domains (Figure 1) [25]. Mutation of the PSAP domain suggested that this domain corresponds to a conventional L domain, and two hybrid analyses revealed that the interaction between PFV Gag and TSG101 is indeed mediated by this sequence. In contrast, the inactivation of PPPI suggested an unconventional mechanism to facilitate PFV egress [25], and mutant viral particles displayed reduced infectivity. Similarly, mutation of the conserved YEIL motif revealed no classical L domain function but resulted in a reduced rate of Gag processing by the viral protease, as well as in the release of noninfectious VLPs [96]. Finally, viruses possessing multiple L domains can also change the L-domain usage to replicate in various cells as M-PMV. Indeed, it was found that the PPxY constitutes a major L-domain in several cell lines, while the PSAP sequence was shown to function as a L-domain in HeLa cells, and it is thought to be dispensable for viral production in 293T and COS-7 cells [23].

## 6. Interplay between L Domains and ESCRT Machinery

**Figure 4:**
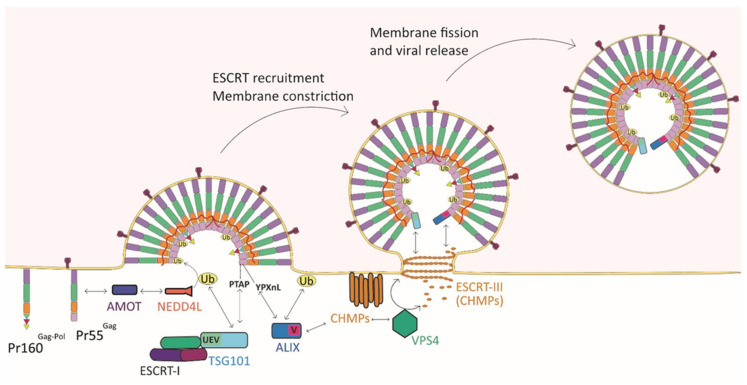
The recruitment of the ESCRT cellular factors by L domains in HIV Pr55^Gag^ ensures viral particles release. The cellular factor angiomotin (AMOT) promotes the recruitment of the ubiquitin ligase NEDD4L at HIV-1 budding sites. The ESCRT machinery is then recruited by NEDD4 family, and this interaction seems to be related to the ubiquitination of viral structural proteins. The P(T/S)AP L domain of Pr55^Gag^ recruits the ESCRT-I factor TSG101 at viral assembly sites by direct binding to its ubiquitin E2 variant (UEV) domain, and the YPXnL motif recruits the ESCRT-III-associated factor ALIX by binding to its V domain. The ESCRT-III proteins drive the interaction with the VPS4 ATPase. This late-acting factor leads to membrane remodeling and its fission, and it drives the disassembling of the ESCRT-III filaments. The process ends with the release of the newly formed viral particle.

Historically, the first in vitro and in vivo evidence that L domains within retroviral Gag interact with host factors involved in the ubiquination machinery were provided by Leis and Carter and Leis labs [61,69,97], thus indicating an important link between this cellular pathway and retroviral budding. Indeed, the NEDD4-like family of E3 ubiquitin protein ligases was found to specifically interact with the RSV PPPPYV L domains [69,97], while the HIV-1 PTAPP L domain binds the homologue of ubiquitin-conjugating (E2) enzyme TSG101 [61]. Viral particle release was then found to be ensured by the specific interaction between L domains and the ESCRT machinery, which is known to remodel membrane. ESCRT proteins have been originally characterized in yeast for their role in multivesicular bodies (MVBs) biogenesis (for a review, see [98]) and are functionally conserved throughout several archaeal species and eukaryotes [99]. In metazoans, it is now well established that ESCRT proteins, beyond their role in the biogenesis of MVBs and in cytokinetic abscission [100,101], are also involved in many remodeling processes, including vesicle and virus budding from PM, endolysosomal membrane and PM repair, neuronal pruning, nuclear envelope maintenance, and autophagy (for a review, see [9]). In humans, the core of the ESCRT machinery is composed of the stalk-shaped hetero-tetramer ESCRT-I (TSG101, VPS28, VPS37A-D, and MVB12/UBAP1), the Y-shaped hetero-tetramer ESCRT-II (two copies of EAP20, one copy of EAP30 and EAP45), the ESCRT-III complex formed by IST1 and the charged multivesicular body proteins (CHMP) 1 to 7 (CHMP1A/B, CHMP2A/B, CHMP3, CHMP4A-C, CHMP5, CHMP6, and CHMP7), the ESCRT-associated protein ALIX, and the AAA ATPase VPS4 (for a detailed list of the homolog components of the ESCRT machinery in yeast, see [10]). The recruitment of the ESCRT factors to their proper sites of action at membranes is enabled by targeting adapter proteins including the HRS/STAM complex (also named ESCRT-0), the centrosomal protein 55 (CEP55), LEM domain 2 protein (LEMD2), and arrestin domain-containing protein 1 (ARRDC1) (for a review, see [9]). Remarkably, P(T/S)AP motifs are used by several of these adapter proteins, including the HRS subunit of ESCRT-0 [102] and ARRDC1 [103], to recruit the ESCRT machinery via the ubiquitin E2 variant (UEV) domain of TSG101. Similarly, the P(T/S)AP L domain in HIV-1 and EBOV recruits the ESCRT-I factor TSG101 at viral assembly sites by direct binding to its UEV domain [61,104,105,106,107]. Moreover, the expression of an N-terminal fragment of TSG101 containing UEV was found to inhibit HIV-1 and FIV release, and in this sense, the FIV PSAP motif behaves similarly to the PTAP motif of HIV-1 [108]. Interestingly, a PTAP motif duplication reported in some HIV-1-infected patients [109,110,111] was associated with an enhanced interaction with TSG101 [112], as well as with an increased retroviral replication fitness and a decreased susceptibility for protease inhibitors [84,112,113]. Other L domains such as the HIV-1 YPXnL recruits the ESCRT-III-associated factor ALIX by direct binding to its V domain [62,79,114,115], while the HTLV-1 PPxY motif binds to the WW domain of members of the HECT E3 ubiquitin ligases NEDD4 family (see Section 6.3) [69,116] (Figure 4). Moreover, if the ESCRT-I and ESCRT-II complexes constitute early acting factors that coordinate ESCRT-III recruitment and membrane curvature, the ESCRT-III polymerization and depolymerization are thought to be the main driving force for membrane remodeling and fission [117,118,119,120,121,122], although the exact mechanism by which these components mediate this process is still unclear. In cytoplasm, ESCRT-III proteins exist in a monomeric closed autoinhibited state [123,124,125] that is overcome by binding with the upstream ESCRT components [126,127,128]. This leads to the membrane binding and to interaction with the VPS4 ATPase that plays a key role in ESCRT-III-mediated membrane scission by remodeling and disassembling ESCRT-III filaments [117,119,129,130,131]. Therefore, VPS4, together with the ESCRT-III complex, corresponds to late acting factors [9] that are usurped by viruses for viral egress.

### 6.1. L Domain Interaction with the ESCRT-I

The role of ESCRT-I in viral egress was exhaustively characterized in the case of HIV-1, and the interaction between two ESCRT-I factors such as the C-terminal domain of TSG101 and the N-terminal domain of VPS28 [132] is thought to be essential to ensure HIV-1 budding. Indeed, fusion of TSG101 mutated in its VPS28 binding domain to an HIV-1 Gag protein deleted of the p6 domain (also named Gag∆p6) [133], as well as the same TSG101 mutants expressed in cells depleted of endogenous TSG101 [134], failed to rescue viral budding. Conversely, fusion of TSG101, VPS37B, or VPS37C to the C-terminus of Gag∆p6 efficiently rescued VLP budding, despite L domain truncation [106,134,135]. In addition, knockdown experiments of TSG101 using siRNAs led to a defective HIV-1 budding, which can be rescued by TSG101 *trans*-complementation [104], while the overexpression of the UEV domain of TSG101 inhibited HIV-1 release [105]. The ESCRT-I UBAP1 factor was also found to promote HIV-1 budding when fused to the C-terminus of a Gag protein lacking L domains, although its depletion by siRNAs did not impact on virus release [136]. Similarly, depletion or overexpression of the ESCRT-I MVB12 had no direct effect on virus egress, even if the infectivity and virion morphogenesis resulted to be defective [137]. Furthermore, depletion of ESCRT-II had little effect on HIV-1 release but blocked the ASV (avian sarcoma virus) budding, and conversely, depletion of ESCRT-I had little effect on ASV release but blocked HIV-1 budding, suggesting that ASV and HIV-1 Gag proteins use different combinations of ESCRT proteins to promote budding [74]. Similarly, RSV and HIV-1 Gag particle release is achieved through independent ESCRT-mediated pathways [138], even though they are linked through TSG101–NEDD4 interaction. However, while the importance of ESCRT-I factors in HIV-1 budding is well established, the role of ESCRT-II factors is yet to be clarified [74,139,140,141,142], and further investigations will then be necessary to fully understand their function in HIV-1 cycle. In the context of HIV-2, TSG101 and Gag precursor (Pr57^Gag^) were also found to interact in vitro and ex vivo via the PTAPP motif in the p6 domain of the precursor (Figure 1) and via the N-terminal Ubc-conjugation homology domain of TSG101. Moreover, the overexpression of TSG101 resulted in an increased level of ubiquitination of Pr57^Gag^, thus indicating the involvement of the cell ubiquitination machinery during the late stages of the viral life cycle [17]. A PSAP L-domain binding TSG101 was also identified in the p2 domain of the FIV Pr50^Gag^ precursor (Figure 1). Interestingly, the Bro1 domain of human ALIX was found to rescue FIV mutants lacking TSG101-interacting motif. However, in contrast to HIV-1, mutations of ZFs in NC domain did not impair FIV rescue, suggesting conserved and divergent mechanisms employed by lentiviruses to achieve viral budding [19].

### 6.2. L Domain Interaction with ALIX

The role of ESCRT-III-associated ALIX protein in YPXnL-mediated viral budding was first determined in the EIAV context, where yeast two hybrid and GST pull down assays showed that the p9 domain of EIAV directly interacts with ALIX, and that this interaction would depend on the YPDL sequence, as substitutions in this motif inhibited the interaction between p9 and ALIX [62,143]. Moreover, the overexpression of truncated ALIX in its Bro1 domain, or the depletion of endogenous ALIX using siRNA knockdown, inhibited p9-mediated EIAV budding. Similar effects were observed for HIV-1 constructs, where p6 domain was replaced by the EIAV p9 domain [143]. However, even though it has been well established that ALIX plays a crucial role in EIAV budding, its function in HIV-1 egress has been formerly considered as secondary. Indeed, the second L domain of HIV-1 (i.e., YPXnL) in p6 was found to provide an alternative pathway for viral egress by interacting with the V-domain of ALIX [62], which in turn leads to the recruitment of ESCRT-III at budding sites via a direct interaction between its Bro1 domain and the C-terminal domain of CHMP4 [114,144]. However, early findings suggested that this interaction plays a minor role in HIV-1 release in comparison to PTAP–TSG101 interaction. Moreover, in contrast to EIAV budding mediated by the p9 domain, depletion of ALIX with siRNA only slightly affected p6-mediated release of HIV-1 from P4/R5 cells, and the overexpression of ALIX truncated in its Bro1 domain did not drastically inhibit HIV-1 p6-mediated budding from P4/R5 or 293T cells [143]. In addition, deletion or mutations of the YPXnL L domain within p6 did not completely impaired HIV-1 budding from COS or HeLa cells [13,58,59,115]. On the other hand, overexpression of the V domain of ALIX considerably inhibited the release of HIV-1 and EIAV virions form HeLa or COS-1 cells [79,115,145], suggesting that HIV-1 release displays cell type dependence [59,146]. This notion was also supported by the fact that although the TSG101-P(T/S)AP interaction has been shown to play a more important role than the ALIX-YPXnL interaction in HIV-1 budding from various epithelial cell lines, YPXnL mutations were found to play a major role in Jurkat T cells [146], and similarly, in HAP1 cells, the YPXnL–ALIX interaction was found to mainly regulate HIV-1 particle production [141].

Moreover, the overexpression of ALIX was shown to rescue the budding of HIV-1 PTAP mutants, and this would depend on the interactions occurring between its Bro1 domain and CHMP4B [114,147]. Moreover, substitutions in the YPXnL motif that abrogates ALIX interaction with Pr55^Gag^ are thought to impair the recruitment of the ESCRT-III machinery, presumably due to catastrophic disassembly of the ESCRT components [148]. Altogether, these findings show the implication of ALIX in the final stages of ESCRT-mediated budding in the HIV-1 context, supporting the idea that ALIX is recruited to the membrane neck at the end of viral particle assembly [149] and mainly acts as a scaffold for ESCRT-III polymerization during viral budding (for a review, see [150]).

As mentioned above, the HIV-1 subtype C (HIV-1C) naturally lacks the YPX_n_L L domain, and in this case, the overexpression of the V domain of ALIX did not affect viral particle release [82]. In this context, the PYXE domain that replaces the YPX_n_L motif in patients in therapy failure [83] was found to interact with ALIX, thus behaving as a L domain to enhance viral budding [84,85]. Similarly, precursors of some simian immunodeficiency virus (SIV) (such as the SIV rhesus macaque and the SIV African green monkeys) lack YPX_n_L motif, although they can bind ALIX. Indeed, crystal structures revealed that in this case anchoring Tyr and nearby hydrophobic residues lead to the interaction between the precursors and the V domain of ALIX, thus revealing how lentiviruses employ diverse sequences to bind ALIX to efficiently promote virus budding [151].

Finally, the suppression of ALIX and TSG101 mediated by RNA interference (RNAi) was also enabled to clarify the contribution of these factors in the production of a DNA virus, the herpes simplex virus type 1 (HSV-1). Indeed, these findings suggested the presence of alternative mechanisms to recruit the ESCRT-III proteins that provide the scission of the membrane and thus the release viral particles [152].

### 6.3. L Domain Interaction with E3 Ubiquitin Ligase NEDD4 Family and the Ubiquitination Machinery

The overexpression of the HECT ubiquitin E3 ligase NEDD4L was observed to stimulate the release of HIV-1 constructs lacking TSG101- and ALIX-binding L domains, increasing infectious titers over 20-fold [153]. Moreover, efficient M-PMV budding was found to be related to the recruitment of NEDD4-like proteins by a PPXY L domain [153], thus indicating an alternative pathway to recruit the ESCRT machinery at viral budding sites [69,116]. The recruitment of the ESCRT machinery by E3 ubiquitin ligases NEDD4 family is linked to the ubiquitination [61,69,154,155,156,157]. Indeed all retroviral Gag polyproteins, except Gag from spumaviruses [158], are ubiquitinated (for a review, see [159]), and ESCRT-0, ESCRT-I, and ESCRT-II recognize and interact with ubiquitylated cargos (for a review, see [160]). Moreover, NEDD4 interacts with the RSV PPxY L domain via its WW domain [69,161], and overexpression of WW domain impaired RSV budding [69], similarly to the mutations occurring in the HECT catalytic site [97]. Likewise, the PPxY motif of HTLV-1 Pr53^Gag^ precursor interacts with the WW domains of several members of NEDD4 family (including NEDD4, WWP1, and ITCH) (for a review, see [161]). The recruitment of NEDD4 by the PPxY L domain was found to promote Pr53^Gag^ ubiquitination, and at this level, TSG101 is then recruited by the second PTAP L domain, thereby ensuring viral budding [95]. Similarly, MLV release is promoted by the interaction occurring between WWP1 ubiquitin ligase and the viral PPxY L domain [116]. Furthermore, the depletion of free cellular ubiquitin by proteasome inhibitors resulted in an inhibited RSV egress, and importantly this phenotype was rescued by ubiquitin overexpression or by ubiquitin fusion to RSV [26]. Interestingly, in the EIAV context, the fusion of ubiquitin to the C-terminus of EIAV Pr55^Gag^ precursor lacking the YPxnL L domain led to overcoming of the release defects [154], thus demonstrating that ubiquitin can functionally counterbalance the absence of retroviral L domains. All these findings confirm that ubiquitination is a part of retroviral budding machinery.

The interaction between PPxY and the WW domain in HECT E3 ubiquitin ligases also regulates the budding of several viruses outside the *Retroviridae*, as for instance rhabdoviruses and filoviruses [162]. These viruses possess a PPxY L domain in their matrix protein (M or VP40, respectively) that functionally interacts with the WW domains of E3 ubiquitin ligases [161,163,164,165,166]. It was shown that VP40 protein of EBOV interacts with WWP1, and its ubiquitination enhances viral budding. Moreover, WWP1 depletion by siRNA was observed to induce the inhibition of VLP budding [167]. Interestingly, although HIV-1 Pr55^Gag^ does not contain a PPxY L domain, the overexpression of catalytically active NEDD4-2 (NEDD4L) E3 ubiquitin ligase can rescue the budding of an HIV-1 construct where both PTAP and YPXnL L motifs were deleted [153,168]. Furthermore, the recruitment of NEDD4L at HIV-1 budding sites was found to be promoted by the cellular factor angiomotin (AMOT) [169] (Figure 4). Finally, other cellular proteins were found to provide an alternative access to ESCRT-III recruitment and act as adaptors, such as, for example, the arrestin-related trafficking (ART) proteins [170].

## 7. Quasi-Enveloped RNA Viruses and the ESCRT Pathway

It was recently shown that several non-enveloped viruses such as hepatitis A virus (HAV), poliovirus, rotavirus, and norovirus [5,6,7] can be referred to as quasi-enveloped viruses since they enclose themselves in vesicles derived from host membranes and exit cells non-lytically (for a review, see [171]). Interestingly, several studies have indicated that the formation of quasi-enveloped HAV virions (eHAV) also depends on the ESCRT components ALIX, VPS4, CHMP2, and IST1 [5,172,173,174]. Notably, one of the HAV capsid major structural proteins VP2 encodes two YPXnL motifs that interact with ALIX [5,172]. Mutational analysis revealed that single substitution of Leu for Ala within either YPXnL motifs resulted in reduction of eHAV release, and that dual substitutions of Leu for Ala in both motifs impaired eHAV budding, but not the assembly of infectious intracellular particles [172]. In those double mutants, the failure in eHAV releasing correlates with the loss of interaction between the capsid and ALIX [172], and similar budding defects were observed when ALIX was depleted by RNAi [5]. Interestingly, eHAV capsid contains a pX domain on its surface carrying several ubiquitination sites that could promote the ESCRT recruitment [171], and pX was found to interact with the V domain of ALIX, thus participating to eHAV biogenesis, although no defined ALIX-binding motif has been identified within pX thus far [174]. Altogether, these findings suggest that HAV recruits ALIX to bud into membranous vesicles, thus facilitating viral protection from neutralizing antibodies [5] and viral spreading as it was demonstrated for rotaviruses, noroviruses, and enteroviruses [6,7].

## 8. Conclusions

Enveloped RNA viruses derive their envelope from the host as they bud through cellular membranes, and viral budding requires the deformation of the cellular membrane followed by a fission event allowing the generation of distinct cellular and viral membranes. Many viruses therefore hijack the cellular ESCRT machinery to remodel the membranes at viral budding sites, thereby enabling the release of infectious enveloped viral particles. To recruit the ESCRT factors, enveloped viruses use conserved viral assembly L domains encoded by their multifunctional structural proteins. Three types of L domains have been characterized thus far: P(T/S)AP, YPXnL, and PPxY, and two other types, FPIV and PLPPV, have been reported in the M protein of paramyxoviruses [72,77,78] and in the MMTV Pr77^Gag^ precursor [73], respectively, even though the cellular partners of these two last motifs remain to be identified. In addition, it was shown that quasi-enveloped viruses, such as HAV, that can exit cells non-lytically within vesicles, encode two YPXnL motifs in their structural protein VP2 that interact with ALIX, and use the ESCRT pathway to promote their budding [5,172,173,174]. As the mechanisms for ESCRT recruitment through the action of L domains and consequent budding are well conserved processes between different RNA virus families, a better comprehension will be determinant for the development of new broad-spectrum antiviral drugs directed against viral release. For example, it was observed that compounds that block the PPxY–NEDD4 interaction also efficiently inhibited the egress of MARV [175,176]. Similarly, recent studies have shown that prazole compounds can inhibit the release of some enveloped viruses, including HIV-1, EBOV, Mayaro virus (MAYV), and HSV by covalently binding the UEV domain of TSG101, which results in the disruption of TSG101–ubiquitin interaction [177,178,179]. Such a class of inhibitors interfering with the budding machinery could thus resultingly be extremely useful for impairing the production of newly emerging RNA viruses for which no therapeutics are available.

## Data Availability

Not applicable.
